# Supplementary material for: Role of Inflammatory and Immune-Nutritional Prognostic Markers in Patients Undergoing Surgical Resection for Biliary Tract Cancers
Source: Cancers (Basel). 2021 Jul 18;13(14):3594. doi: 10.3390/cancers13143594 (PMC8305862; doi:10.3390/cancers13143594)
Supplement: Supplementary file 1 [file cancers-13-03594-s001.zip › cancers-1253042-supplementary.pdf]

cancers

Supplementary Materials: Role of Inflammatory and Immune-Nutritional Prognostic Markers in Patients Undergoing Surgical Resection for Biliary tract Cancers

Simone Conci, Tommaso Campagnaro, Elisa Danese, Ezio Lombardo, Giulia Isa, Alessandro Vitali, Ivan Marchitelli, Fabio Bagante, Corrado Pedrazzani, Mario De Bellis, Andrea Ciangherotti, Alfredo Guglielmi, Giuseppe Lippi and Andrea Ruzzenente

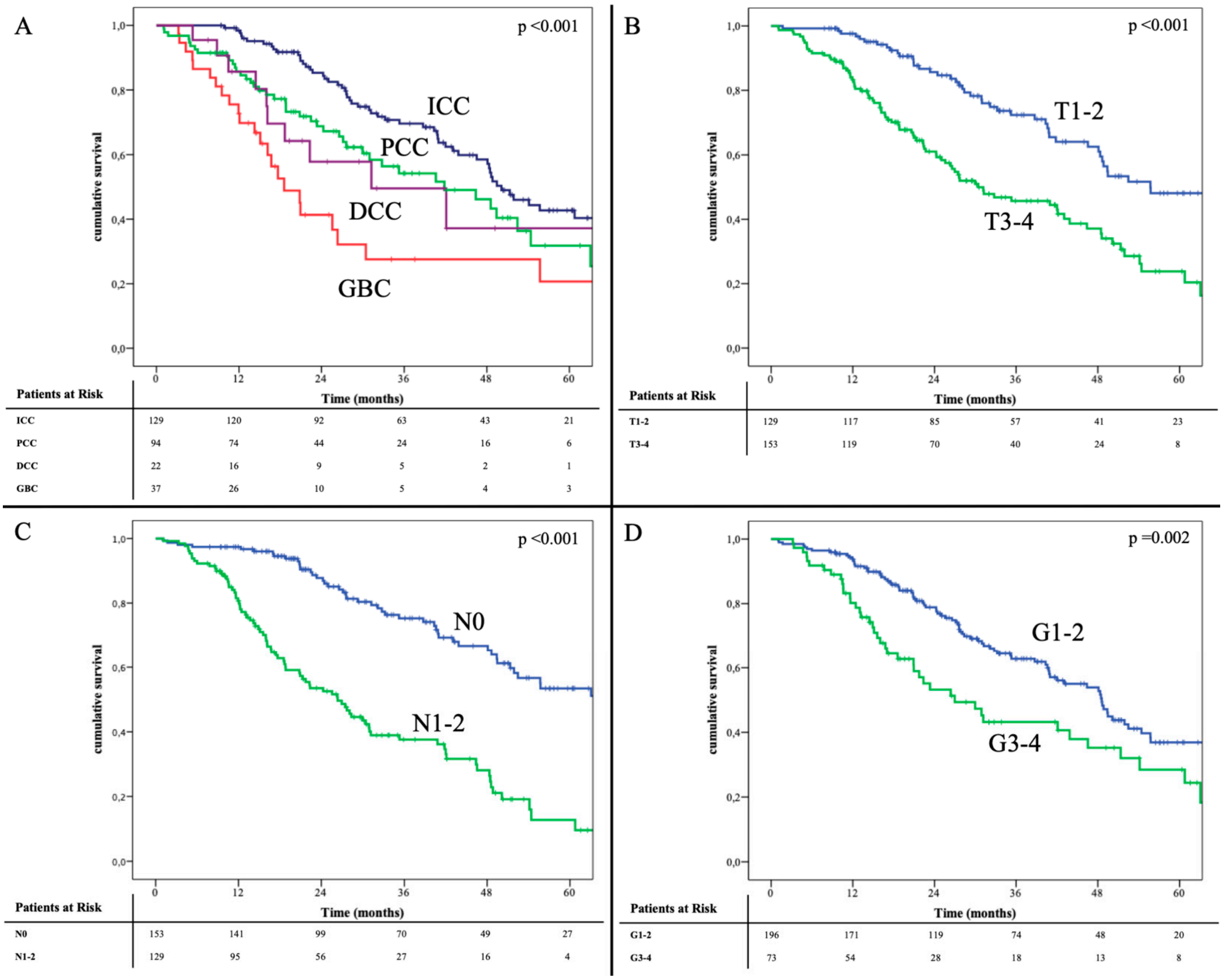

Figure S1. Survival curves according to the main clinical and pathological factors: **A**. Type of Biliary Tract Cancer (BTC); **B**. AJCC 8thEd. T Stage; **C**. AJCC 8thEd. N Stage; **D**. Histologic grading. ICC, intrahepatic cholangiocarcinoma; PCC, perihilar cholangiocarcinoma; DCC, distal cholangiocarcinoma; GBC, gallbladder cancer; AJCC, American Joint Committee against Cancer.

Table S1. Comparison of Clinical and pathological characteristics according to the type of BTC.

Characteristics		ICC n 129	PCC n 94	DCC n 22	GBC n 37	*p* Values
Age, years, median (IQR)		68.9 (59.9–74.4)	69.6 (63.4–74.3)	75.6 (67.1–78.1)	68.1 (61.2–74.7)	0.032
Gender, male, *n* (%)		73 (56.6)	64 (68.1)	14 (63.6)	16 (43.2)	0.057
CA 19-9, U/mL, median (IQR)		58.0 (15.0–411.0)	483.0 (91.5–2656.2)	115.0 (45.0–1304.0)	302.0 (30–5785.7)	0.003
Jaundice, *n* (%)		11 (8.5)	83 (88.3)	19 (86.4)	14 (37.8)	< 0.001
PBD, *n* (%)		8 (6.2)	70 (74.5)	21 (95.5)	14 (37.8)	< 0.001
Preop Chemotherapy, *n* (%)		8 (6.2)	3 (3.2)	0 (0.0)	1 (2.7)	0.685
PVE, *n* (%)		2 (1.6)	7 (7.4)	0 (0.0)	0 (0.0)	0.086
Tumor size, mm, median (IQR)		50 (40–70)	30 (20–43)	20 (10–20)	40 (30–60)	< 0.001
Major hepatectomy, *n* (%)		78 (60.4)	79 (84.0)	0 (0.0)	7 (18.9)	< 0.001
Biliary resection, *n* (%)		38 (29.5)	94 (100.0)	22 (100.0)	14 (37.8)	< 0.001
Vascular resection, *n* (%)		5 (3.9)	19 (20.2)	1 (4.5)	3 (8.1)	0.001
AJCC 8th T Stage, *n* (%)	T1–2	77 (59.7)	40 (42.6)	1 (4.5)	11 (29.7)	< 0.001
	T3–4	52 (40.3)	54 (57.4)	21 (95.5)	26 (70.3)	
AJCC 8th N Stage, *n* (%)	N0	88 (68.2)	45 (47.9)	7 (31.8)	13 (35.1)	< 0.001
	N1–2	41 (31.8)	49 (52.1)	15 (68.2)	24 (64.9)	
Histologic grading, *n* (%)	G1–2	98 (76.0)	68 (72.3)	10 (45.5)	20 (54.0)	0.004
	G3–4	29 (22.5)	19 (20.3)	12 (54.5)	13 (35.2)	
	n/a	2 (1.5)	7 (7.4)	0 (0.0)	4 (10.8)	
Macrovascular invasion, *n* (%)		19 (14.7)	43 (45.7)	3 (13.6)	9 (24.3)	< 0.001
Microvascular invasion, *n* (%)		78 (60.5)	78 (83.0)	14 (63.6)	29 (78.4)	0.010
Radicality, *n* (%)	R0	89 (69.0)	62 (66.0)	15 (68.2)	27 (73.0)	0.883
	R1	40 (31.0)	32 (34.0)	7 (31.8)	10 (27.0)	
Severe Morbidity, *n* (%)		23 (17.8)	29 (30.9)	4 (18.2)	6 (16.2)	0.048
Post-operative mortality, *n* (%)		0 (0.0)	3 (3.2)	0 (0.0)	2 (5.4)	0.016
Hospital stay, days, median (IQR)		8 (7–13)	16 (12–27)	18 (14–35)	9 (5–24)	< 0.001
Postop Chemotherapy, *n* (%)		101 (78.3)	48 (51.1)	14 (63.6)	18 (48.6)	< 0.001

ICC; intrahepatic cholangiocarcinoma; PCC; peri-hilar cholangiocarcinoma; DCC; distal cholangiocarcinoma; GBC, gallbladder cancer; IQR, interquartile range; PBD, preoperative biliary drainage; PVE, portal vein embolization;

Table S2. Comparison of distribution of Inflammatory-based and nutritional prognostic scores according to the type of BTC.

Prognostic Score		ICC n 129	PCC n 94	DCC n 22	GBC n 37	p values
GPS, *n* (%)	0	111 (86.1)	44 (46.8)	13 (59.1)	24 (64.9)	
	1	16 (12.4)	32 (34.0)	4 (18.2)	11 (29.7)	<0.001
	2	2 (1.5)	18 (19.1)	5 (22.7)	2 (5.4)	
mGPS, *n* (%)	0	114 (84.4)	60 (63.8)	14 (63.6)	26 (70.3)	
	1	13 (10.1)	16 (17.1)	3 (13.7)	9 (24.3)	<0.001
	2	2 (1.5)	18 (19.1)	5 (22.7)	2 (5.4)	
PI, *n* (%)	0	104 (80.6)	58 (61.7)	13 (59.1)	25 (67.6)	
	1	14 (10.1)	29 (30.9)	7 (31.8)	8 (21.6)	0.015
	2	11 (8.5)	7 (7.4)	2 (9.1)	4 (10.8)	
NLR, median (IQR)		2.71 (1.97–3.89)	3.29 (2.47–4.56)	3.06 (1.69–4.13)	3.14 (1.96–3.76)	0.026
PLR, median (IQR)		147.0 (110.2–180.5)	198.9 (136.9–275.7)	135.8 (95.9–214.2)	148.5 (116.4–190.5)	0.001
LMR, median (IQR)		3.46 (2.56–4.90)	2.98 (1.97–4.12)	3.19 (2.39–3.81)	3.55 (2.33–5.27)	0.197
PNI, median (IQR)		52.4 (47.7–55.1)	45.1 (39.6–49.5)	47.3 (41.7–51.3)	49.8 (44.8–53.5)	<0.001

GPS, Glasgow Prognostic Score; mGPS, modified Glasgow Prognostic Score; PI, Prognostic Index; NLR, neutrophil to lymphocyte ratio; PLR, platelet to lymphocyte ratio; LMR, lymphocyte to monocyte ratio; PNI, prognostic nutritional index.

Table S3. Mortality according to the different inflammatory and immune-nutritional markers. GPS, Glasgow Prognostic Score; mGPS, modified Glasgow Prognostic Score; PI, Prognostic Index; NLR, neutrophil to lymphocyte ratio; PLR, platelet to lymphocyte ratio; LMR, lymphocyte to monocyte ratio; PNI, prognostic nutritional index.

Prognostic Marker		Univariate Analysis		Logistic regression		
		n (%)	*p* values	OR	95% C.I.	*p* values
GPS	0	1/192 (0.5)	0.037	8.884	0.978-20.661	0.052
	1–2	4/90 (4.4)				
mGPS	0	2/214 (0.9)	0.093	4.892	0.800-19.913	0.086
	1–2	3/68 (4.4)				
PI	0	2/200 (1.0)	0.149	3.759	0.616-22.929	0.151
	1–2	3/82 (3.7)				
NLR	<3.13	3/146 (2.1)	0.533	0.711	0.117-4.324	0.712
	≥3.13	2/136 (1.5)				
PLR	<178.2	3/174 (1.7)	0.635	1.075	0.177-6.542	0.937
	≥178.2	2/108 (1.9)				
LMR	≥3.47	3/126 (2.4)	0.400	0.532	0.088-3.237	0.494
	<3.47	2/156 (1.3)				
PNI	≥48.6	1/154 (0.6)	0.018	8.547	1.876-13.789	0.005
	<48.6	4/128 (3.1)				
